# Implications of Disability for Functional Trajectory, Service Use, and Expenditures

**DOI:** 10.1093/geroni/igab046.599

**Published:** 2021-12-17

**Authors:** Wayne Anderson, Gretchen Alkema

## Abstract

People with disabilities face a diverse array of health care and support needs. These needs can vary by disability type, degree, and timing of the advent of functional limitations. These differences have implications for needed health care service use and related expenditures. The symposium will open with a Centers for Disease Control and Prevention-sponsored analysis of adult disability-associated health care expenditures, both nationally and by U.S. state, in total, by per adult, by per adult with disability, and by payer, to illustrate the contribution and variation of these expenditures to individual states and the health care system. We will next present a U.S. Department of Health and Human Services’ Office of the Assistant Secretary for Planning and Evaluation effort to identify onset and patterns of reduced functional ability at end of life for older adults with and without dementia as related to other comorbidities. The last paper will present a Commonwealth Foundation study on older adults with functional disabilities and multiple chronic conditions, comparing those with high health care needs versus the subset of those people who are also high cost. Patterns of utilization differed between these two groups, and by state. These findings have implications for the development of care models that might best meet people’s needs. Our discussant will respond to the studies’ findings and discuss the important role that efforts to understand the nature of disability and functional status and the scale and scope of service use and costs have for people with disabilities.

